# Trends in US Hematology/Oncology Physician Perceptions and Referral Practices for Hematopoietic Cell Transplant: A National Survey Conducted by NMDP

**DOI:** 10.1002/cam4.71551

**Published:** 2026-01-29

**Authors:** Aye Mon Thida, Ankita Shah, Kelley Frederick (Steffens), Samantha Watters, Mingqian Duan, Steven Devine, Susan T. Vadaparampil, Joseph Pidala

**Affiliations:** ^1^ NewYork‐Presbyterian‐Weill Cornell Medical Center New York city New York USA; ^2^ NMDP Minneapolis Minnesota USA; ^3^ CIBMTR (Center for International Blood and Marrow Transplant Research), NMDP Minneapolis Minnesota USA; ^4^ Department of Health Outcomes and Behavior Moffitt Cancer Center Tampa Florida USA; ^5^ Department of Blood and Marrow Transplantation and Cellular Immunotherapy Moffitt Cancer Center Tampa Florida USA

**Keywords:** access, allogeneic, barrier, hematologic malignancies, hematopoietic‐cell transplantation, underserved populations

## Abstract

**Background:**

To evaluate trends in allogeneic hematopoietic cell transplant (HCT) referral perceptions and practices, we compared surveys of Hematology‐Oncology (Hem‐Onc) physicians conducted in 2015 (*N* = 150), 2019 (*N* = 302), and 2024 (*N* = 183) and reported changes over time.

**Methods:**

Eligible participants included Hem‐Onc physicians in the United States seeing at least 10 hematologic malignancy patients in the last year. Questions covered perceptions of HCT, referral practices, perceived barriers to referral, and endorsement of HCT education and patient support resources.

**Results:**

There were positive trends in HCT perceptions, increased expected HCT benefit, and positive outcomes with older AML patients. Overall reported disease‐specific referral rates increased over the survey periods. Participants reported earlier HCT referral timing across the survey time periods for all diagnoses, as well as increased referral of AML in first complete remission (vs. later stages of disease). While reported barriers to HCT referral persist, 2024 responses (vs. 2019) had significant reduction in concern over finding a suitable HCT donor, adverse post‐HCT outcomes, patient age, and medical comorbidities or psychosocial barriers to HCT. Across all hematologic malignancies in 2024, the average maximum referral age was 73.5 years. Respondents indicated a strong desire for additional physician education and patient‐level support.

**Conclusions:**

We identified positive trends in HCT perceptions and referral practices, and reduced barriers. Encouragingly, these trends suggest that evidence surrounding HCT benefit and broadened eligibility for HCT are reaching the larger Hem‐Onc community, and this may address historical barriers to access. Ongoing education and outreach are needed to facilitate additional progress.

## 1 Introduction

Allogeneic hematopoietic cell transplant (HCT) is a well‐established, potentially curative therapy for patients with hematologic malignancies and disorders. Despite advances that have made HCT possible for more patients, safer, and improved long‐term outcomes, HCT is underutilized with persistent access disparities [1, 2, 3, 4, 5, 6, 7]. Many patients with otherwise HCT‐eligible diagnoses are never referred for HCT consult, [8, 9, 10, 11, 12] and a fraction of referred patients successfully reach HCT [1, 2, 3, 4, 6].

Hematopoietic cell transplant (HCT) is performed in academic tertiary care centers, as the delivery of HCT care is complex and highly specialized. Accordingly, practicing Hematology/Oncology (Hem‐Onc) physicians, whether in community practices or academic hospitals, act as key partners in access to this specialized care, either choosing to refer patients for HCT consult or not. Prior physician surveys have identified patient‐, provider‐, and system‐level barriers to HCT referral, as well as potential educational and outreach efforts to address this problem [13].

Hematopoietic cell transplant (HCT) technology and supportive care continue to improve [14] and several important developments may have impacted HCT physicians’ perceptions and referral practices. Highly impactful publications supporting HCT as a curative strategy have saturated the Hem‐Onc field more broadly [15, 16, 17], and support that alternative HCT donor types can overcome the historic race/ethnicity‐based barrier to HCT dependent on matched donor availability [18, 19, 20, 21]. Additionally, risk‐adapted HCT approaches have reduced barriers based on chronological age or medical comorbidities [22, 23, 24]. Educational and outreach efforts may have influenced attitudes of referring physicians and reduced systemic barriers to access. There has been increasing attention on the importance of health disparities, including disparate access to care as a key driver of observed health disparities.

To address this important issue, a contemporary study is needed to evaluate current knowledge and practices of Hem‐Onc providers toward HCT referral. We leveraged three sequential NMDP^SM^ (formerly the National Marrow Donor Program/Be the Match) physician surveys to examine changes in HCT perceptions and referral practices over time.

## 2 Methods

### 2.1 Design of Sequential Physician Surveys

The current analysis explored trends in U.S. Hem‐Onc physician responses across three sequential online cross‐sectional surveys conducted in 2015, 2019, and 2024. Each survey was conducted as a single time point cross‐sectional design, and the current trend analysis was not planned a priori. Survey respondents were asked to report on their perceptions and referral practices for HCT over the preceding year.

Each survey represented a cross‐sectional study design with convenience sampling through different national online panel sources made up of physicians who had signed up to participate in paid research studies. In each wave, physicians specializing in Heme‐Onc were invited to participate in the survey through email invitations sent by the panel sources. Complete details about the recruitment methodology are available in Table S1.

The 2015 survey was conducted from June through July 2015. Resolution Research, a market research agency, provided full‐service research support including recruitment and data analysis. NMDP performed the research with grant support from the National Comprehensive Cancer Network (NCCN) and Pfizer. A $150 honorarium was provided to participants. Survey completion was projected to require up to 30 min. Eligible participants were board‐certified U.S.‐based Hem‐Oncs with adult or adult/pediatric practices who did not personally perform allogeneic HCT and saw at least 10 patients with acute myelogenous leukemia (AML), myelodysplastic syndrome (MDS), acute lymphoblastic leukemia (ALL), multiple myeloma (MM), Non‐Hodgkin lymphoma (NHL), or other hematologic malignancies/aplastic anemia over the past year. Purely pediatric‐focused clinicians were not included, given anticipated differences in incidence of the studied hematologic malignancies in that setting vs. adults, as well as potential differences in HCT indications and referral practices in the pediatric setting. A maximum of 50 participants seeing fewer than 2 AML patients per year (new consults and/or follow‐up) were allowed to complete the survey, given AML‐focused survey content. A maximum of 20 with less than 1 year of experience in practice post‐fellowship were allowed to participate. A total of 150 physicians completed the survey. Participant identities were kept anonymous from NMDP, and participants were informed that the research was funded through a grant from NCCN/Pfizer. NMDP was not identified as a stakeholder of the research.

The 2019 survey was conducted from September through October 2019. NMDP contracted Survey Healthcare Globus, a healthcare market research panel provider, to recruit physician participants. A $50 honorarium was provided to participants by Survey Healthcare Globus. The survey was projected to require approximately 6 min for completion. Eligible participants were board‐certified U.S.‐based Hem‐Oncs with adult or adult/pediatric practices who did not personally perform allogeneic HCT and saw at least 10 patients with AML, MDS, or ALL over the past year. A maximum of 100 participants seeing fewer than 2 AML patients per year (new consults and/or follow‐up) were allowed to complete the survey. A maximum of 20 with less than 1 year of experience in practice post‐fellowship were allowed to participate. A total of 302 physicians completed this survey. Participant identities were kept anonymous from NMDP, and participants were unaware that NMDP had commissioned the research.

The 2024 survey was conducted from June through July 2024. NMDP contracted MedSurvey, a healthcare market research panel provider, to recruit physician participants. A $100 honorarium was provided to participants by MedSurvey. The survey was projected to require approximately 11 min for completion. Eligible participants were board‐certified U.S.‐based Hem‐Oncs with adult or adult/pediatric practices who did not personally perform allogeneic HCT and saw at least 10 patients with AML, MDS, ALL, severe aplastic anemia, bone marrow failure, or myelofibrosis over the past year. A maximum of 100 participants seeing fewer than 2 AML patients per year (new consults and/or follow‐up) were allowed to complete the survey. A maximum of 20 with less than 1 year of experience in practice post‐fellowship were allowed to participate. A total of 183 physicians completed the survey. Participant identities were kept anonymous from NMDP, and participants were unaware that NMDP had commissioned the research.

### 2.2 Shared and Unique Survey Content

Given the current study was not planned a priori, we report here the shared content across survey iterations that facilitated the analysis versus unique content that was not compared. To organize this shared content, we first describe shared survey domains, and then shared questions within these domains: The core domains consistently represented across all three surveys (2015, 2019, and 2024) include: (I) Clinical Practice Setting, (II) Referral Practice, (III) Current Perceptions of Allogeneic Transplant for AML, (IV) Post‐transplant Care, and (V) Demographics. Each domain included questions grouped by a common conceptual focus (for example, referral decision‐making or transplant perceptions), with 100% agreement in the inclusion of these themes across the surveys, enabling cross‐survey comparisons (see Data S1). Notably, the “Tools and Resources” domain was included only in the 2015 and 2024 surveys, while “Current Perceptions of Allogeneic Transplant for All Diseases” was limited to the 2015 survey.

The Clinical Practice Setting domain contained seven shared questions (Table S2), Referral Practice included six shared questions alongside additional non‐shared items (Table S3A,B), and Perceptions of Allogeneic Transplant for AML featured six shared questions with further non‐shared inquiries (Table S4A,B). Post‐transplant Care had four shared questions and additional non‐shared questions (Table S5A,B), while Demographics comprised three shared questions, supplemented by nine non‐shared questions in the 2024 survey (Table S6A,B). The Tools and Resources domain, assessed in 2015 and 2024, had no shared questions (Table S7A–C), and Perceptions of Allogeneic Transplant for All Diseases, captured solely in 2015, provided broader insights into transplantation perspectives (Table S8).

### 2.3 Analysis Methods

Descriptive statistics were used to summarize and report data on the survey elements studied. These were compared in an exploratory analysis across the three survey iterations where possible based on shared survey items. In 2024, survey respondent subgroups were defined by referral rate as High > 66% referral rate amongst usual patients seen within past year, Medium: 34%–65%, and Low: < 33%, as well as years in practice (high experience: > 21 years in practice, medium: 11–20 years, and low: < 10 years). Subgroups based on awareness of NMDP (referring provider self‐reported awareness of the organization) were also explored. Pairwise comparisons of proportions were performed using the Chi‐square test. To control for the inflation of type I error, we applied the Benjamini–Hochberg procedure to adjust *p*‐values for false discovery rate (FDR). All analyses were performed using R version 4.5.1.

## 3 Results

### 3.1 Survey Respondent Demographics

A comparative summary of the survey respondent demographics across the 2015, 2019, and 2024 iterations is presented in Table 1. Table S9 presents the additional respondent demographic questions included in the 2024 survey, as well as patient demographics. Notably, across the surveys, there was greater representation of university/academic affiliation providers (2015: 15%, 2019: 15%, 2024: 33%), and a corresponding decrease in private practice (59%, 58%, 33% respectively), while most respondents specialized in both oncology and hematology (2015: 91%, 2019: 95%, and 2024: 94%). Years in practice were similar across the 2015, 2019, and 2024 surveys (21+ years in practice: 31%, 28%, 28%; 11–20 years: 33%, 38%, 34%; and 10 or fewer years: 36%, 34%, 38%).

**TABLE 1:** Comparative summary of survey respondent demographics across 2015, 2019, and 2024 surveys.

	2024	2019	2015
	(*n* = 183)	(*n* = 302)	(*n* = 150)
**Medical specialty**			
Oncology	8 (4%)	13 (4%)	13 (9%)
Hematology	3 (2%)	2 (1%)	1 (1%)
Oncology and Hematology	172 (94%)	287 (95%)	136 (91%)
**Practice setting**			
University/academic‐based or affiliated	60 (33%)	63 (21%)	30 (20%)
Hospital or health system‐based practice	62 (34%)	63 (21%)	32 (21%)
Group private practice	57 (31%)	160 (53%)	76 (51%)
Solo private practice	4 (2%)	16 (5%)	12 (8%)
**Years in practice**			
0–10 years	70 (38%)	104 (34%)	54 (36%)
11–20 years	62 (34%)	114 (38%)	50 (33%)
21+ years	51 (28%)	84 (28%)	46 (31%)
Median	14	15	14
**Patient ages treated**			
Adult	160 (87%)	282 (93%)	129 (86%)
Adult and pediatric	23 (13%)	20 (7%)	21 (14%)
**Mean annual patient volumes by disease (total seen in Hem‐Onc practice)**			
ALL	14	14	13
AML	22	21	15
MDS	30	30	**25**
**Mean annual patient referral volumes by disease (number referred for HCT consult)**			
ALL	9	8	5
AML	12	11	6
MDS	9	8	5
**Percent of time spent in clinical care**			
< 25%	1 (1%)	2 (1%)	2 (1%)
25%–49%	2 (1%)	6 (2%)	6 (4%)
50%–75%	20 (11%)	21 (7%)	7 (5%)
> 75%	160 (87%)	273 (90%)	135 (90%)
**Number of hem‐onc physicians in your practice**			
Solo practice	5 (3%)	16 (5%)	11 (7%)
2–5 physicians	41 (22%)	96 (32%)	63 (42%)
6–10 physicians	62 (34%)	95 (31%)	47 (31%)
More than 10	75 (41%)	95 (31%)	29 (19%)
**Practice location**			
Rural/township	14 (8%)	26 (9%)	11 (7%)
Small town	15 (8%)	24 (8%)	13 (9%)
Suburban	68 (37%)	122 (40%)	66 (44%)
Urban	86 (47%)	130 (43%)	60 (40%)

**FIGURE 1:**
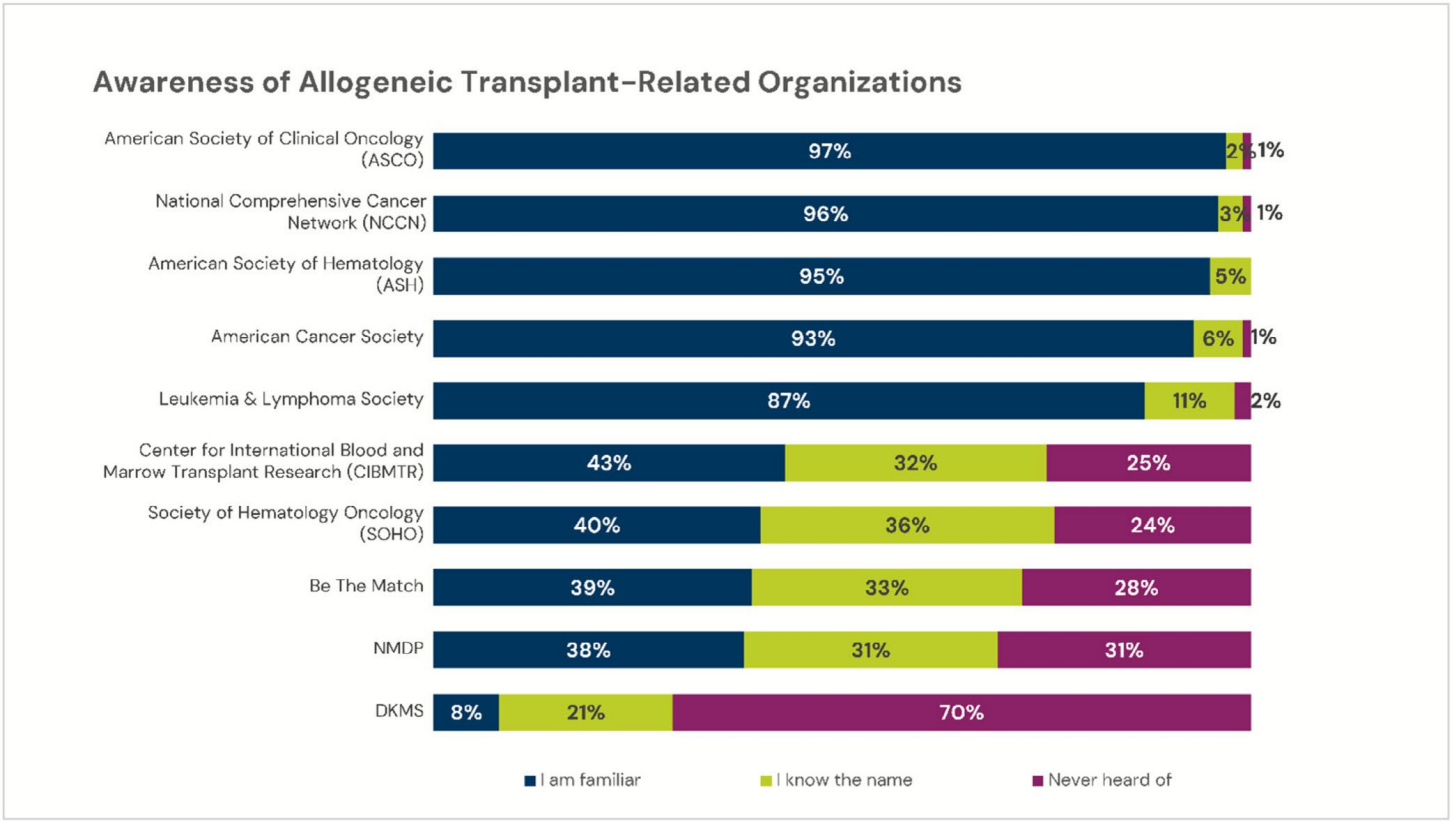
Proportion of 2024 survey respondents who are familiar with various HCT‐relevant organizations. *N* = 183.

Respondents indicated that post‐HCT care (following first 3 months post‐HCT) was largely provided by the HCT center (80%, 67%, 72% for 2015, 2019, 2024 respectively), while interestingly some also indicated they (49%, 46%, 48%) or primary care physician (9%, 4%, 5%) contributed to this post‐HCT care (response included all that apply). The 2024 survey respondents ranked their awareness of major organizations relevant to HCT. The proportion indicating they were familiar with each organization is shown in Figure 1. Differences were observed when responses were stratified by high/medium/low referral groups: percentage familiar for NMDP (high 44%, medium 46%, low 25%), Be The Match (high 41%, medium 51%, low 25%), and CIBMTR (high 51%, medium 51%, low 26%).

### 3.2 Perceptions of HCT

**FIGURE 2:**
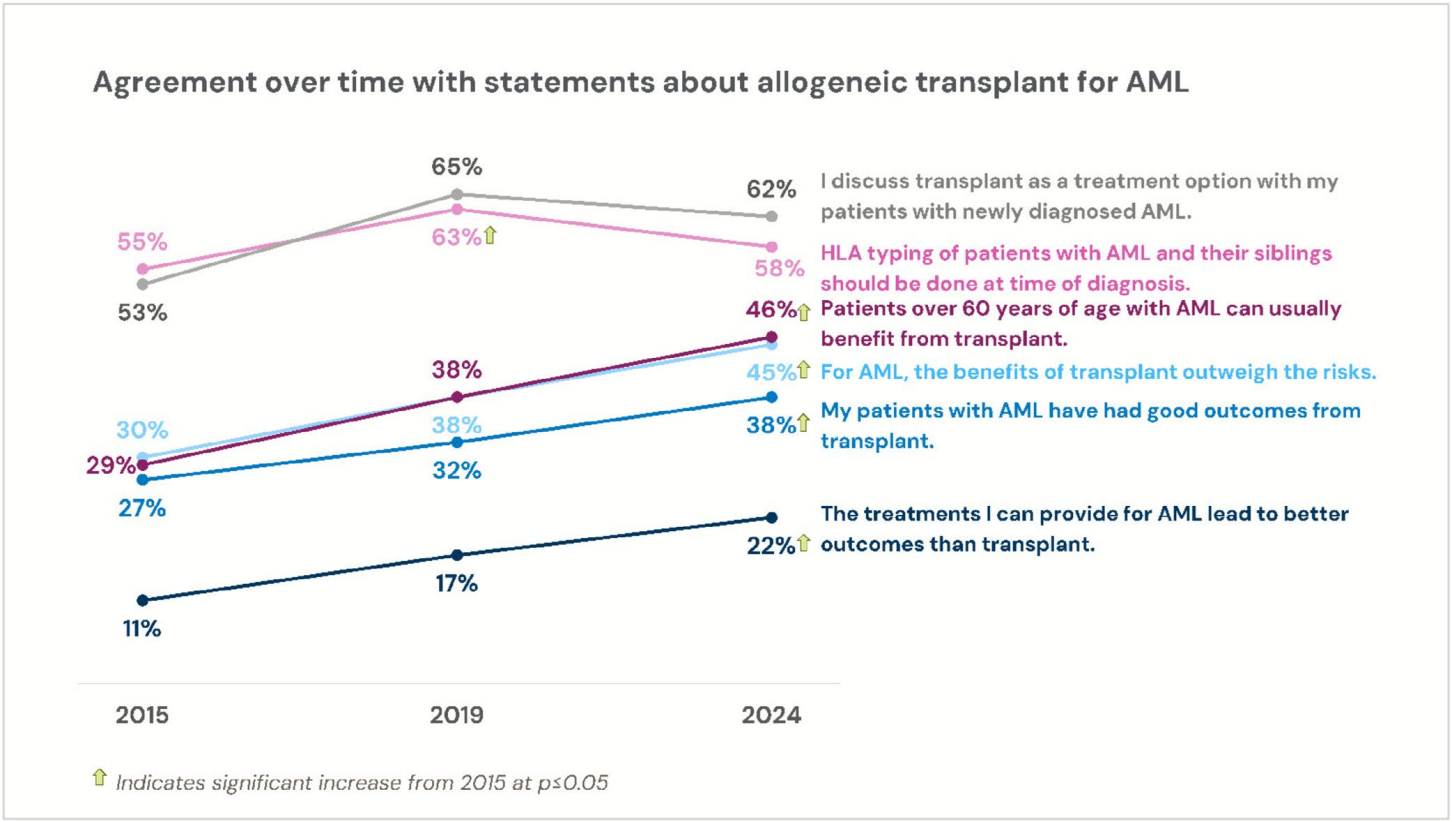
The proportion of respondents agreeing with AML perception statements across 2015, 2019, and 2024 surveys. Agreement defined as rating 8–10 on a 1–10 scale (1 = Strongly disagree, 10 = Strongly agree). Increases or decreases from the 2015 survey (*p* < 0.05) are noted with arrows. *N* = 150 for 2015, *N* = 302 for 2019, and *N* = 183 for 2024.

Across the 2015, 2019, and 2024 surveys, positive trends were observed regarding several HCT perception items related to AML. Percentages of physicians reporting high agreement with statements related to AML treatment options, HCT outcomes, HLA typing, and patient age are reported in Figure 2. When the 2024 survey respondents were stratified by awareness of NMDP (aware vs. not aware), more positive perceptions of HCT were observed (percent supporting each comparing aware vs. non‐aware groups): good outcomes after HCT (54% versus 28%), HCT benefit outweigh risk (57% versus 40%), patients over 60 benefit from HCT (53% versus 44%), HLA typing should be done at diagnosis (70% versus 58%), and discussing HCT as treatment option at AML diagnosis (70% versus 60%). Amongst those aware versus. unaware of NMDP, 46% versus. 21% were academically affiliated, respectively.

### 3.3 Overall and Disease‐Specific Referral Rates

Total self‐reported referral rates (percent referred for HCT/total patients seen with HCT indication) increased across the survey time points of 2015, 2019, 2024: 32%, 35%, 38%, respectively. The total reported referral rates increased with fewer years of practice experience (21+ years: 34%, 11–20 years: 36%, 0–10 years: 43%). Interestingly, the majority of 2024 survey respondents (78% in total, 40% indicating no change vs. 38% indicating increase) reported that they expected their own HCT referral rates would not change or increase over the next 5–10 years. In contrast, 22% anticipated their HCT referral rates would decrease over that time frame.

**FIGURE 3:**
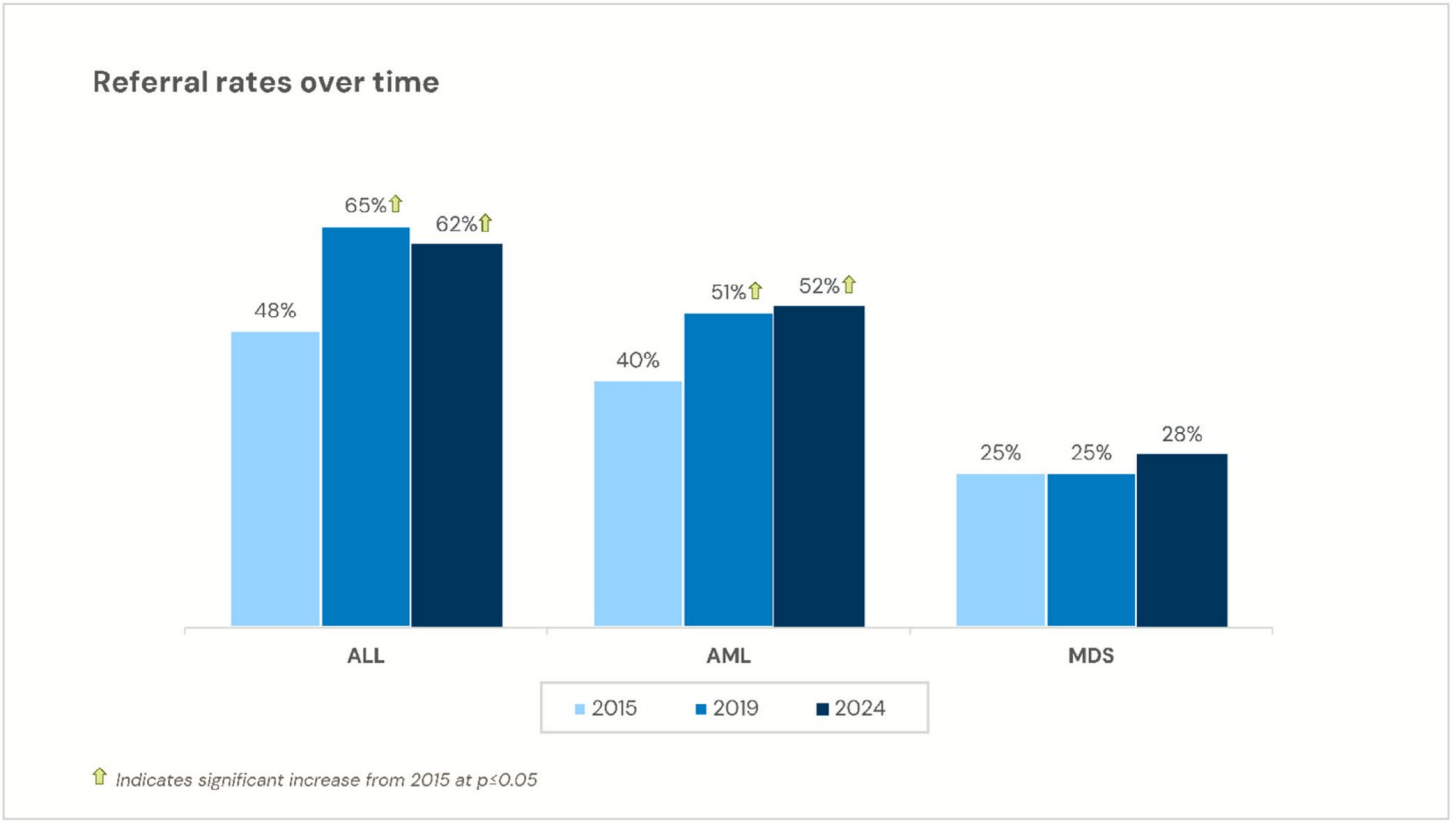
Referral rate changes over time from 2015, 2019, and 2024 surveys by disease. Increases or decreases from the 2015 survey (*p* < 0.05) are noted with arrows. *N* = 149 for 2015, *N* = 302 for 2019, and *N* = 183 for 2024. The self‐reported referral rate represented the total number of referred patients versus the total number of patients seen with the disease.

The disease specific referral rates reported (percent referred for HCT/total number of patients seen with each disease) differed. For example, in 2024, reported referral rates differed across this disease spectrum: ALL 62%, severe aplastic anemia 60%, AML 52%, bone marrow failure 51%, myelofibrosis 33%, MDS 28%. Positive trends were observed in disease‐specific reported referral rates across the 2015, 2019, 2024 surveys considering the HCT indications of ALL and AML, yet limited change was noted for MDS, as shown in Figure 3. Disease specific reported referral rates also differed based on NMDP awareness: Specific HCT referral rates reported by subjects in the 2024 survey, for those aware of NMDP versus not, were 64% versus 59% for ALL, 58% versus 49% for AML, 32% versus 23% for MDS, 68% versus 62% for SAA, 63% versus 55% for bone marrow failure syndromes, and 35% versus 34% for myelofibrosis.

**FIGURE 4:**
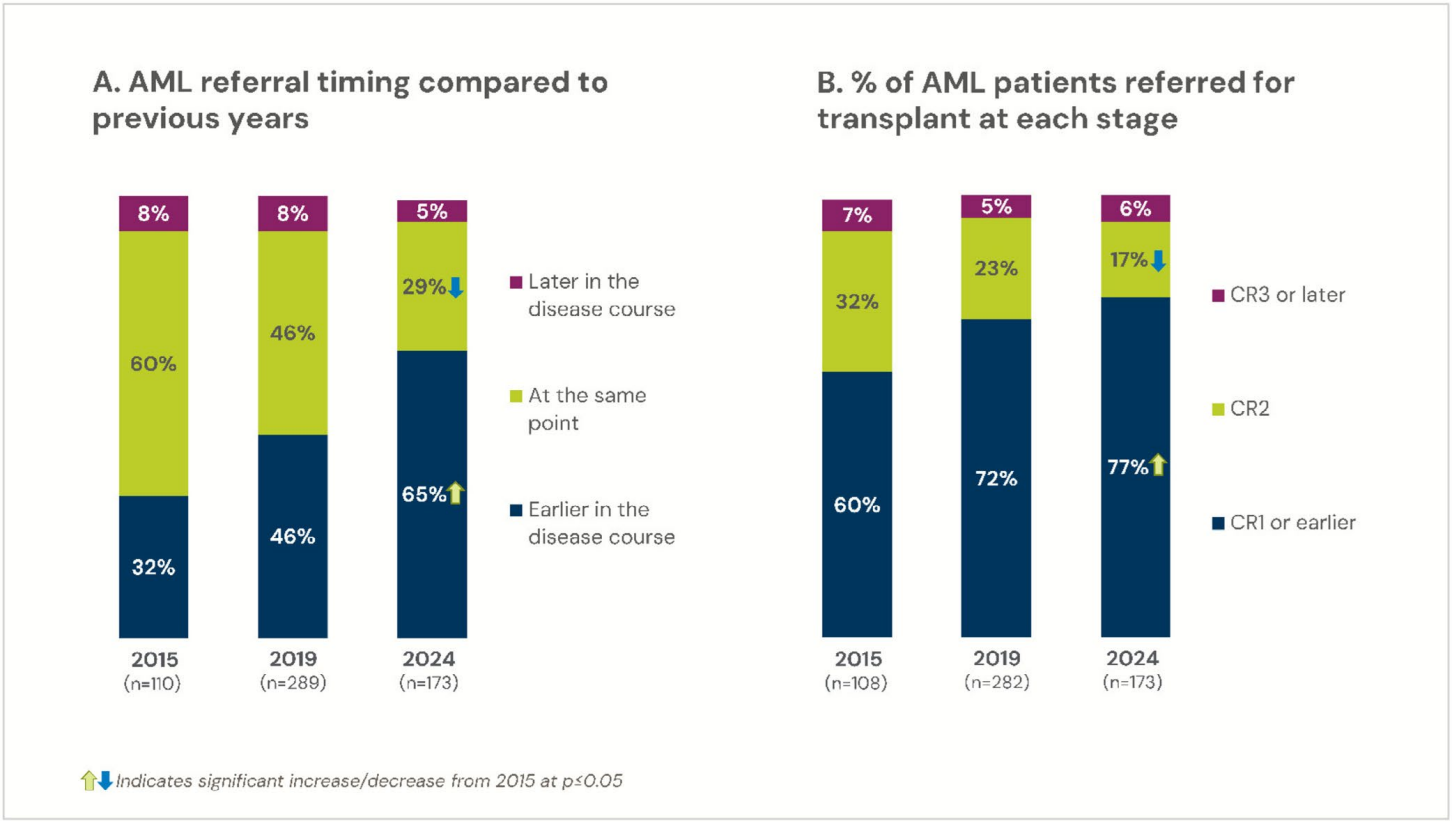
(A) Referral timing by year across 2015, 2019, and 2024 surveys for AML. Physicians reported how the timing of their referrals within the past 12 months compares to previous years. (B) The percentage of patients referred for AML at each stage of complete remission. Self‐reported referral percentages are represented as the total number of patients referred versus the total number of patients seen.

Referral timing was also explored through asking respondents how the timing of HCT referral within the past year compared to prior years overall and for disease specific categories. The data supported favorable trends over the 2015, 2019, and 2024 surveys respectively, as shown in Figure 4A,B.

### 3.4 Reasons for HCT Non‐Referral

**FIGURE 5:**
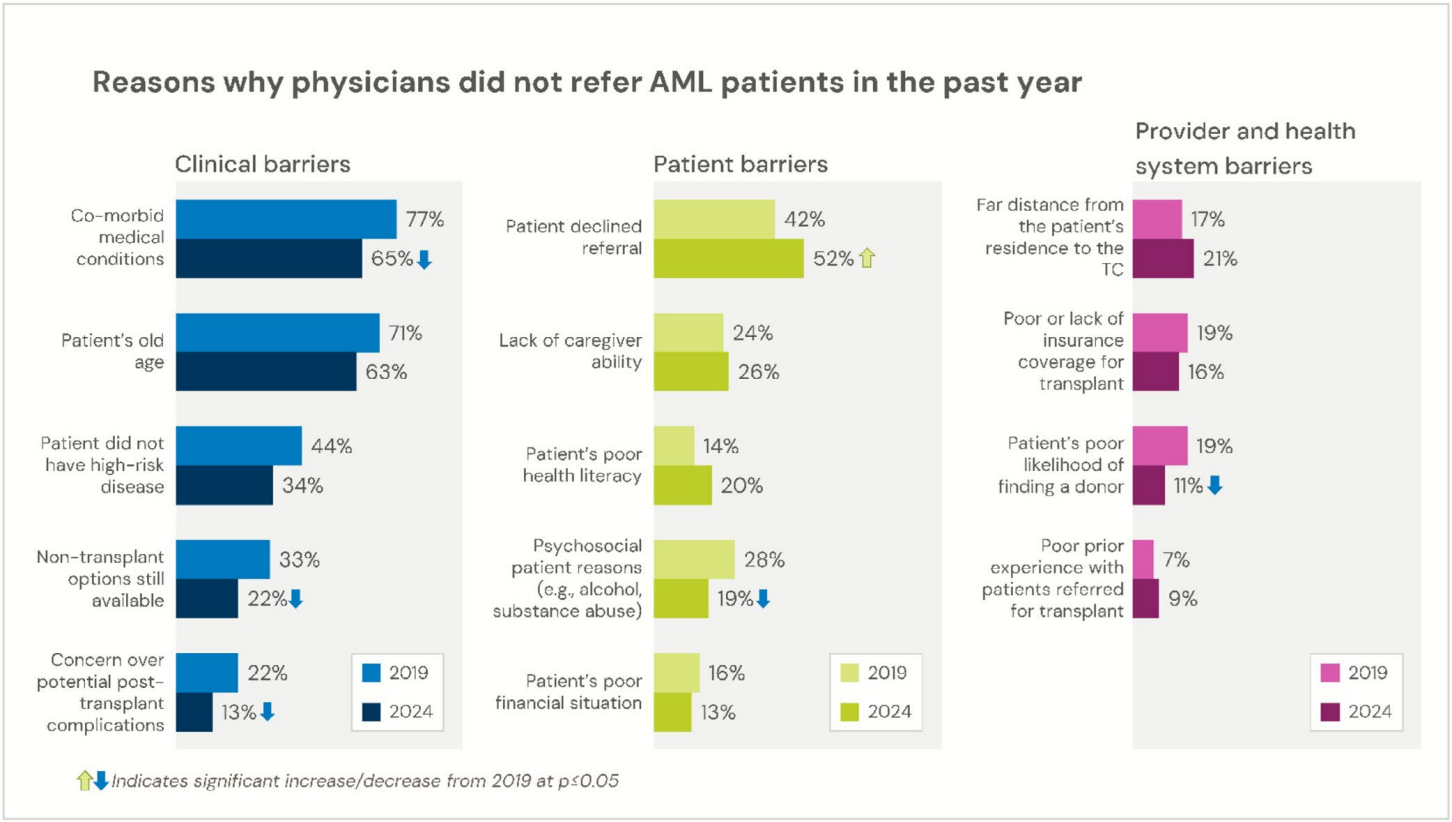
Barriers to referral reported for AML patients. Top reported reasons for non‐referral are reported for 2019 and 2024, with significant differences (*p* < 0.05) indicated by arrows. *N* = 269 for 2019 and *N* = 152 for 2024.

Respondents in the 2019 and 2024 surveys indicated which factors (multiple selections possible) were involved in no HCT referral for their AML patients. The listed options in the surveys were broadly focused on patient‐level factors (patient age, comorbid medical or psychosocial issues, patient health literacy), disease risk (patient does not have high‐risk disease), donor factors (patient likelihood of finding a donor), systemic factors (caregiver availability, distance from HCT center, insurance coverage for HCT, patient financial situation), and patient or provider decision‐making (patient declined HCT referral, non‐HCT treatment options available, concern over post‐HCT complications, prior experience with HCT referrals, concern over post‐HCT complications). These factors were cited by survey respondents as reasons for non‐referral of AML patients and are listed in Figure 5.

### 3.5 Endorsement of Education and Support Resources

**FIGURE 6:**
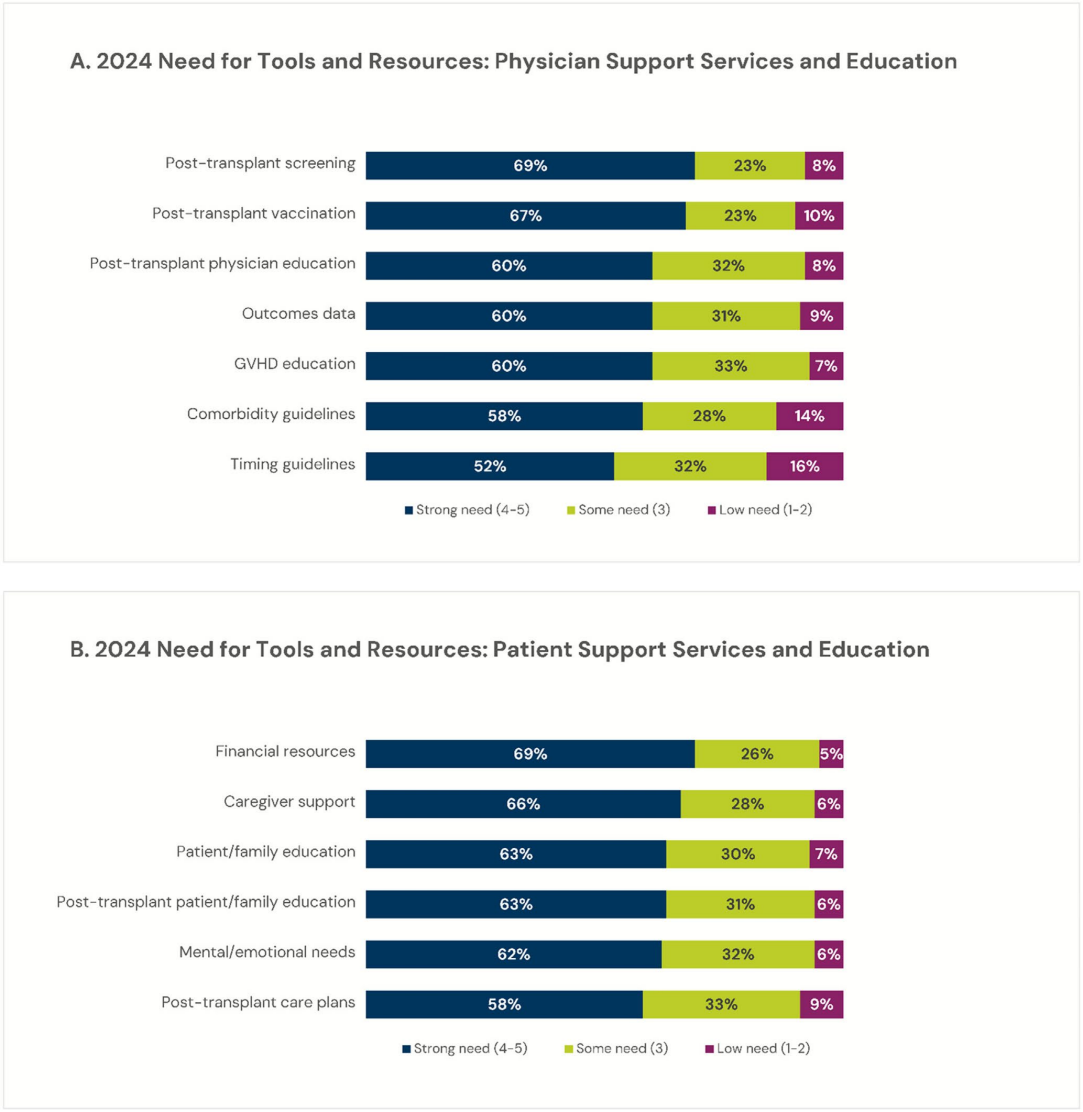
(A) 2024 physician survey respondent self‐reported need for tools and resources related to physician support services and education. (B) 2024 physician survey respondent self‐reported need for tools and resources related to patient support services and education *N* = 183.

Amongst 2024 survey respondents, there was generally a desire to learn more about managing post‐HCT patients as well as an indication that additional education is needed: A total of 54% of respondents reported that they were interested in learning more about post‐HCT care (while 26% indicated moderate support of this statement, and only 20% disagreed). Only 37% reported that they have the information they need to provide post‐HCT care, and only 32% indicated they had adequate training in post‐HCT care. When split by length of time in practice, minimal differences were seen in the proportion indicating they agree with each statement regarding post‐HCT care (by 0–10 years, 11–20 years, > 21 years, total respectively): interested in learning more (51%, 55%, 55%, 54%), have information needed (30%, 35%, 47%, 37%), and had adequate training (29%, 32%, 37%, 32%). Figure 6A displays that 2024 respondents indicated a strong desire for additional resources on Hem‐Onc physician education and post‐HCT care, while Figure 6B shows significant need for patient‐level support.

Amongst 2015, 2019, and 2024 survey respondents, there was stability in the belief that transplant centers are the primary provider of post‐HCT care. The majority of respondents felt that after the first 2 months post‐transplant, the transplant center still primarily provides post‐HCT care for patients, either alone or in combination with Hem‐Onc physicians and/or primary care physicians: 80% (2015), 67% (2019), and 72% (2024), note that percentages reported were not mutually exclusive. Less respondents felt that the primary provider of post‐HCT care was the Hem‐Onc physician either alone or in combination with the transplant center and/or primary care physician: 49% (2015), 46% (2019) and 48% (2024), note that percentages reported were not mutually exclusive.

## 4 Discussion

It is well established that many patients with HCT eligible hematologic malignancies and disorders are not referred for HCT consultation, thus missing an opportunity at a potentially curative therapy. Prior work has suggested patient‐, provider‐, and system‐level factors that contribute to this problem, including prior surveys of practicing Hem‐Onc physicians [25, 26]. Several factors may have positively influenced awareness and perceptions of HCT referral over time, but whether this has directly influenced HCT referral practices remains understudied. To address this need, we leveraged a series of three sequential Hem‐Onc physician surveys to examine trends over time.

The results generally suggest that awareness and perceptions of HCT have moved in positive directions over the nearly 10‐year period covered by these data. Improvements were seen in self‐reported willingness to discuss HCT as a treatment option with AML patients early in their disease course, and perceptions of HCT's benefit improved, including for those most commonly affected by AML (age > 60). These improvements suggest that providers have greater familiarity both with HCT as part of the treatment trajectory for these patients and the benefit of HCT for older patients. Importantly, respondents also reported that they had personally observed good post‐HCT outcomes for their referred patients, which likely shapes their willingness to consider this as a curative therapy for current and future patients. The proportion supporting the concept that AML patients and their siblings should be HLA typed at diagnosis remained relatively stable over time; however, the exact reasons for this are unclear. For example, the survey question did not further delve into whether HLA typing should be done at all, specifically at the time of diagnosis, or at later stages of the disease course. Similarly, the survey did not directly ask about resources to obtain HLA typing, or how certain initiatives (for example, HLA Today services provided by NMDP) to get typing done quickly and without cost to the patient would influence adoption of early HLA typing. These questions deserve further study.

Impressively, the sequential survey results signal enhanced referral rates for ALL and AML. Those Hem‐Onc physicians with fewer years of practice experience reported greater referral rates, suggesting that those with more recent training may be better attuned to the importance of HCT referral. However, there clearly remains significant room for improvement across the board. At best, the overall reported HCT referral rate was 38% for all conditions, and by 2024 only 28% of MDS, 52% of AML, and 62% of ALL cases were reported to be referred for HCT consultation. This highlights an ongoing disconnect between primary Hem‐Onc providers’ perspectives of eligibility vs. HCT physicians who generally prefer early evaluation of all patients with HCT‐relevant diagnoses. We also highlight in particular that there was not a significant improvement in reported referral rate for MDS over the surveys conducted. This is a notable gap, given recent data demonstrating survival advantage for HCT vs. no HCT in MDS [15], as well as publication of important HCT outcome data for MDS according to patient age [27]. The results suggest additional study is needed to verify current MDS referral attitudes and behaviors in a larger set of referring Heme‐Onc providers. Importantly, however, survey items examined referral rates overall per condition, and did not ask nuanced questions to dissect referral rates according to disease risk categories for each (for example, considering cytogenetic or molecular risk stratification tools) or nuanced subgroup‐specific questions regarding patient‐level subgroups (for example, per age, comorbidities, fitness, performance status). This level of detail is critical in practice, and yet not feasible to examine in full in a national physician survey. Greater examination of these nuances, including in focus group‐based discussions, would likely facilitate greater understanding. We do acknowledge, however, that the referral timing data suggest positive trends in referral earlier (for example, first complete remission for AML) in a patient's disease course.

The data on reasons for non‐HCT referral largely recapitulate prior work in the field, where patient age and comorbidities are dominant, with concerns regarding psychosocial factors, caregiver support, and logistical constraints including distance to the HCT center influencing referral decision making. Our comparison from 2019 to 2024 survey data suggests there are some evident positive changes. For example, patient age and comorbidities have decreased as barriers, and similarly the maximum allowable age for HCT referral as a barrier declined over the survey series. These results support at least the possibility that data surrounding the safe use of HCT in older adults or those with comorbidities (for example, through reduced intensity conditioning) have influenced the perceptions and practices of Hem‐Onc physicians. We also observed that concerns over poor post‐HCT outcomes and finding a suitable donor for patients have declined, again potentially demonstrating that advances in alternative donor use and improvements in post‐HCT outcomes may have reached referring Hem‐Onc providers. We acknowledge that these data points do not definitively support these conclusions, and that further study is needed. Encouragingly, however, we observed that referring providers are interested in learning more and are supportive of educational opportunities that could further enhance their understanding of HCT referral timing and post‐HCT outcomes, as well as interventions that could assist patients and caregivers going through this process.

There remain provider and patient challenges in shifting care from HCT centers back to the community following HCT [28]. Among survey respondents for 2015, 2019, and 2024, there is currently an understanding that post‐HCT care falls under the primary responsibility of the HCT center either alone or in combination with hem‐onc physicians and primary care providers. This is coupled with survey data from 2019 and 2024 that highlights that hem‐onc physicians feel they don’t have adequate training in post‐HCT care. Interestingly, the 2024 survey also highlighted hem‐onc respondents have a strong desire for provider‐level educations support and tools. This suggests the interest that hem‐onc physicians have in improving care coordination and becoming more involved and comfortable with patient follow‐up care post‐HCT. Interventions and programs (i.e., NMDP's Pathways to Transplant Program) that help connect hem‐oncs to these educational resources and enhance hem‐onc and HCT physician relationships could help address this need. NMDP's Pathways to Transplant Program ensures hem‐onc access to updated and research driven consultation and post‐transplant care guidelines accessible on their website and app [29]. As such, identifying dissemination channels and implementation strategies for these and other similar resources deserve continued focus.

While the survey explored physician‐level perceptions of referral patterns, it was interesting to note the hem‐onc perspective on whether patients were declining referral to consult. While all other reasons for non‐referral were generally decreasing, more physicians reported patients declining consult as a top reason for non‐referral. The reasons for this could be multifactorial and this was not further assessed within the surveys. It is known that patients face a multitude of challenges including psychosocial, caregiver, and financial challenges that could influence their willingness to move forward with HCT. However, full education on HCT risk/benefit/alternatives can be best facilitated through an HCT consult. This highlights a need to encourage early referral of patients to HCT consultation allowing adequate time to discuss in further detail.

As limitations of this work, most importantly we acknowledge that this trend analysis was not pre‐planned and not formally powered for comparisons. Thus, the positive changes noted over time are considered descriptive and exploratory only. Additionally, no consistent representation of individual physicians is expected over time (across survey iterations); therefore, we can’t assume any individual physician‐level change was captured in these comparisons. Moreover, the study did not account for potential clustering of multiple respondents from the same institution, which may introduce bias due to shared institutional practices. We also note the overall low response rate for each iteration, and that the respondent pool may be biased by known and unknown factors that may have influenced the responses. Importantly, those physicians not represented in these study populations may have even lower HCT referral rates or more adverse HCT perceptions, but this remains unknown based on these data. We also note that the final survey items represent a compromise to keep the survey less time‐intensive for physicians, somewhat at the expense of complete coverage on these important topics. As stated above, there are multiple patient‐level nuances in routine clinical practice that were not covered in detail, such as disease risk assessment tools. More detailed targeted surveys or focus groups may be needed to address these varied clinical scenarios more completely.

## 5 Conclusion

Overall, our data provides high‐level insights into HCT referral attitudes and practices, supporting positive trends over time that may assist in more equitable HCT access for eligible patients.

## Author Contributions

A.M.T., A.S., K.F.S., S.W., M.D., S.D., S.T.V., and J.P. contributed to study design, data analysis, manuscript writing, and are fully responsible for the content of the manuscript.

## Ethics Statement

The study was approved by the NMDP IRB.

## Consent

All survey participants consented to the research participation through an introductory consent question in the survey questionnaires.

## Conflicts of Interest

The authors declare no conflicts of interest.

## Supporting information


**Data S1:** Supporting Information.

## Data Availability

The authors verify that they will consider data requests from interested parties. Any data sharing would require formal data sharing agreements.
