# Supplementary material for: Trends in US Hematology/Oncology Physician Perceptions and Referral Practices for Hematopoietic Cell Transplant: A National Survey Conducted by NMDP
Source: Cancer Med. 2026 Jan 29;15(2):e71551. doi: 10.1002/cam4.71551 (PMC12853074; doi:10.1002/cam4.71551)
Supplement: Supplementary file 1 — Data S1: Supporting Information. [file CAM4-15-e71551-s001.docx]

**Supplemental Table 1: Recruitment Methodology for Surveys Conducted in 2015, 2019, and 2024**

|  | **2024** | **2019** | **2015** |
| --- | --- | --- | --- |
| **Recruitment Agency** | **MedSurvey** | **Survey Healthcare Global** | **Resolution Research** |
| **Panel verification process** | Panelists locked to NPI records, two-factor authentication for identity-linked data, and real-time credential verification | Double opt-in email, public medical directory check, and physical credential verification | Not available |
| **Funding source** | Pro-bono from MedSurvey | Internal NMDP funds | Grant from NCCN/Pfizer |
| **Inclusion criteria for survey invite** | Physicians specializing in hematology/oncology | | |
| **Recruitment methodology** | All studies used convenience sampling via online recruitment panels. All respondents had previously opted in to these research panels to participate in paid research studies. | | |
| **Invite methodology** | Email invitations sent by panel company to opted-in panelists. | | |
| **Anonymity** | Double-blind: in all studies, participant identities were kept anonymous from NMDP and participants were unaware that NMDP sponsored the research. | | |
| **Fielding dates** | June 14 - July 6, 2024 | September - October 2019 | June - July 2015 |
| **Honoraria** | $100 | $50 | $150 |
| **Survey length** | 11 minutes | 6 minutes | 30 minutes |
| ***Number of physicians invited to complete the survey** | 9,000-10,000 | 5,800 | 3,000 |
| **Number who began the survey** | 487 | 539 | Unavailable |
| **Number screened out** | 220 | 200 | Unavailable |
| **Number of completed responses** | 183 | 302 | 150 |
| **Participation rate**  *(% of those invited who clicked into the survey)* | 5% | 9% | Unavailable |
| **Response rate**  *(% of those invited who qualified for and completed the survey)* | 2% | 5% | 5% |
| **Incidence rate**  *(% of respondents who click into the survey and pass the screening criteria)* | 50% | 56% | Unavailable |
| **Physician overlap** | There is no way to confirm whether a physician may have participated in 2 or more waves of the surveys because participants were anonymous, and different panel recruiting agencies were used for each wave. | | |

*Each recruitment agency provided their best estimate for the total number of physicians invited to complete the survey, as this is not a metric that is typically tracked and reported back to clients.

**Supplemental Table 2: Shared Questions in Clinical Practice Setting Across 2015, 2019, and 2024 Surveys**

| **I. Screener - Clinical Practice Setting** | **Year Comparison** | | |
| --- | --- | --- | --- |
|  | **2024** | **2019** | **2015** |
| 1. Medical specialty | x | x | x |
| 2. Board certified or board eligible? | x | x | x |
| 3. Practice setting | x | x | x |
| 4. Years in practice | x | x | x |
| 5. Patient group | x | x | x |
| 6. Transplant performed | x | x | x |
| 7. Number of patients seen | x | x | x |
|  | | | |
| 1. What is your medical specialty?  - Oncology only  - Hematology only  - Oncology and hematology  - Other (Terminate) | | | |
| 2. Are you currently board certified or board eligible in your specialty?  - Yes - No (Terminate) | | | |
| 3. What type of organization best describes your primary practice setting?  - University-based  - University-affiliated community group practice  - Group private practice  - Solo private practice  - Government/VA hospital (Added in 2024) | | | |
| 4. How many years have you been in practice, post-fellowship? | | | |
| 5. For which of the following patient groups do you provide care?  - Adult only  - Pediatric and adult  - Pediatric only (Terminate) | | | |
| 6. Transplant performed | Do you personally perform any of the following?  - Allogeneic hematopoietic cell transplant HCT (Terminate) - Autologous hematopoietic cell transplant HCT - CAR-T therapy | Do you personally perform allogeneic and/or autologous transplants?  (Terminate if YES) | Do you personally perform allogeneic and/or autologous transplants? (Terminate if YES) |
| 7. How many patients have you **seen** in the last 12 months for each of the diseases below? Please provide your best estimate.  (If total <10, terminate.) | - ALL  - AML  - MDS  - Severe aplastic anemia  - Bone marrow failure syndromes  - Myelofibrosis | - ALL  - AML  - MDS | - ALL  - AML  - MM  - MDS  - NHL  - Other hematologic malignancies and aplastic anemia |

**Supplemental Table 3A: Shared Questions in Referral Practice Across 2015, 2019, and 2024 Surveys**

| **II. Referral Practice** | **Year Comparison** | | |
| --- | --- | --- | --- |
|  | **2024** | **2019** | **2015** |
| 1. Patients referred - past 12 months | x | x | x |
| 2. Change in referral timing | x | x | x |
| 3. AML referral stage | x | x | x |
| 4. Reasons for AML non-referral | x | x | x |
| 5. Max age referral | x | x | x |
| 6. Referral by disease criteria | x | x | x |
|  | | | |
| 1. How many patients (newly diagnosed and existing) for whom you delivered care, have you personally referred for transplant consultation in the last 12 months for each of the diseases below? | - ALL  - AML  - MDS  - Severe aplastic anemia  - Bone marrow failure syndromes  - Myelofibrosis | - ALL  - AML  - MDS | - ALL  - AML  - MM  - MDS  - NHL  - Other hematologic malignancies and aplastic anemia |
| 2. How does the timing of your referral for transplant consultation within the past 12 months compare to previous years for each of the following diseases?  - Earlier in the disease course  - At the same point in disease course  - Later in the disease course | - ALL  - AML  - MDS | - ALL  - AML  - MDS | - ALL  - AML  - MDS  - MM  - NHL |
| 3. Of the patients with AML you referred for transplant consultation, at what point in the AML disease course was each patient?  (Question asking number of patients in each option.) | - Prior to first complete remission (CR1)  - First complete remission (CR1)  - Second complete remission (CR2)  - Third complete remission (CR3) or later | - First complete remission (CR1)  - Second complete remission (CR2)  - Third complete remission (CR3) or later | - First complete remission (CR1)  - Second complete remission (CR2)  - Third complete remission (CR3) or later |
| 4. Please indicate whether each of the factors below contributed to your decision not to refer at least one of those AML patients in the past 12 months.  (multi-select check boxes) | - Patient’s old age  - Patient’s poor health literacy  - Patient’s poor financial situation  - Poor or lack of insurance coverage for transplant  - Far distance from patient’s residence to transplant center  - Patient’s poor likelihood of finding a donor  - Psychosocial patient reasons (e.g., alcohol, substance abuse)  - Lack of caregiver ability (e.g., not available, unable to provide care)  - Co-morbid medical conditions  - Patient did not have high-risk disease  - Concern over potential post-transplant complications, including graft versus host disease (GVHD)  - Poor prior experience with patients referred for transplant  - Non-transplant treatment options still available  - Patient declined referral  - Other (Specify) | Same with 2024 | 2015 asked not only for the number of AML patients but also for all other patients that did not get transplant: with these additional reasons:  - Too early in disease course  - Patient had advanced disease  - Rapid disease progression, relapse or patient died  - Patient declined or chose another treatment  - Medical necessity criteria for transplant consultation not met  - Inadequate insurance coverage for donor search or transplant  - Donor not available  - Patient lacked adequate caregiver support  - Other non-medical patient reasons, please specify:  - Other reason, please specify: |
| 5. Max age referral | Is there a maximum adult patient age that you will refer to a transplant center for HCT consultation? Consider any hematologic diseases.  - Yes (please enter the maximum age in years): [RANGE: 18-120]  - No maximum age | What is the maximum adult patient age you will refer for consultation for allogeneic related-donor transplant?   What is the maximum adult patient age you will refer for consultation for allogeneic unrelated-donor transplant? (any hematologic diseases) | What is the maximum adult patient age you will refer for consultation for allogeneic related-donor transplant?   What is the maximum adult patient age you will refer for consultation for allogeneic unrelated-donor transplant? (any hematologic diseases) |
| 6. Referral by disease criteria  For adult patients with AML who have the following disease features or cytogenetics, please indicate if you would refer the patient for allogeneic HCT consultation (assume no other patient barriers):  - Yes  - No  - Don’t know  - Don’t test for this (Only in 2015) | - Primary induction failure  - Measurable residual disease after initial therapy  - CR1  - CR2 or beyond (not previously evaluated for HCT)  - Antecedent hematological disease (e.g., MDS)  - Treatment-related leukemia  - First relapse  - Favorable risk AML  - Intermediate risk AML  - Poor risk AML | - t(8;21)q(22;q22.1): RUNX1-RUNX1T1  - Mutated NPM1 without FLT3-ITD or with FLT3-ITD low  - Mutated NPM1 and FLT3-ITD high  - Wild-type NPM1 without FLT3-ITD or with FLT3-ITD low (without adverse-risk genetic lesions)  - del(5q); -7; -17/abn(17p)  - Wild-type NPM1 and FLT3-ITD high  - Mutated TP53 | - Monosomal karyotype on cytogenetics (e.g., -5, -7)  - Complex karyotype (≥3 cytogenetic abnormalities)  - Good risk karyotype (t (8;21); inv 16; t (16;16)) with C-KIT mutation  - Normal karyotype with no other molecular abnormalities  - Normal karyotype with FLT3-ITD mutation  - Normal karyotype with FLT3-TKD mutation  - Normal karyotype with NPM1 mutation (without FLT3-ITD mutation) |

**Supplemental Table 3B: Non-Shared Questions in Referral Practice Across 2015, 2019, and 2024 Surveys**

| **Non-shared Questions in 2024** | 1. How do you expect the percentage of patients you refer for HCT consultation to change over the next 5-10 years?  - Decrease significantly  - Decrease somewhat  - No change  - Increase somewhat  - Increase significantly  2. Please provide additional details about why you think the percentage of patients you refer for HCT consultation will [pipe in the prior answer] in the next 5-10 years. |
| --- | --- |
| **Non-shared Question in 2019** | 1. Have you referred patients with hematologic malignancies for CAR-T therapy?  - Yes  - No |
| **Non-shared Questions in 2015** | 1. How many patients with AML were referred for transplant consultation in CR2 or later for each of the reasons below?  (Number of patients for each of the options below)  - Patient cytogenetics or molecular abnormalities did not warrant referral in CR1  - Rapid disease progression or relapse post initial response  - Patient declined transplant referral in CR1  - Risk of transplant at CR1 too high given patient’s age or comorbidities  - I recommended other treatment modalities that were better options in CR1  - Insurance authorization or medical necessity criteria not met for consultation earlier in disease course  - Other non-medical patient reasons, please specify:  2. How many of those patients went on to receive a transplant?  (Number of patients for AML versus all other patients for each of the options below)  - You referred for transplant  - Received a transplant  - Didn’t get a transplant  - Not sure |

**Supplemental Table 4A: Shared Questions in Perceptions of Allogeneic Transplant for AML Across 2015, 2019, and 2024 Surveys**

| **III. Current Perceptions of Allogeneic Transplant for AML** | **Year Comparison** | | |
| --- | --- | --- | --- |
|  | **2024** | **2019** | **2015** |
| 1. Over 60 can benefit | x | x | x |
| 2. AML good outcomes | x | x | x |
| 3. My treatments are better | x | x | x |
| 4. Benefits outweigh risks | x | x | x |
| 5. Discuss transplant | x | x | x |
| 6. HLA typing at diagnosis | x | x | x |
|  | | | |
| Please indicate how strongly you agree or disagree with the following statements regarding allogeneic transplant and outcomes of transplant for AML only.  Strongly disagree (1), 2…9, Strongly agree (10) | | | |
| 1. Patients over 60 years of age with AML can usually benefit from transplant | | | |
| 2. My patients with AML have had good outcomes from transplant | | | |
| 3. The treatments I can provide for AML lead to better outcomes than transplant | | | |
| 4. For AML, the benefits of transplant outweigh the risks | | | |
| 5. I discuss transplant as a treatment option with my patients with newly diagnosed AML | | | |
| 6. HLA typing of patients with AML and their siblings should be done at time of diagnosis | | | |

**Supplemental Table 4B: Non-Shared Questions in Perceptions of Allogeneic Transplant for AML Across 2015, 2019, and 2024 Surveys**

| **Non-shared Questions in 2024** | Please indicate how strongly you agree or disagree with the following statements regarding allogeneic transplant and outcomes of transplant for AML only. Strongly disagree (1), 2…9, Strongly agree (10).   1. I/my practice has a strong relationship with the nearest transplant center(s) 2. For AML, I prefer to refer patients to a tertiary cancer center with a dedicated leukemia and/or bone marrow transplant (BMT) program immediately upon diagnosis rather than personally treat them |
| --- | --- |
| **Non-shared Questions in 2019** | 1. If a free service were available that provided high resolution HLA typing through returning an easy-to-use HLA typing kit (buccal swab), how likely would you be to use it for AML patients at the time of diagnosis?   - Definitely would use  - Probably would use  - Might or might not use  - Probably would not use  - Definitely would not use   1. Why do you say you would or would not use this service if it was available? (Open-ended) |
| **Non-shared Questions in 2015** | Please indicate how strongly you agree or disagree with the following statements regarding allogeneic transplant and outcomes of transplant for AML only. Strongly disagree (1), 2…9, Strongly agree (10).   1. Transplant outcomes for AM are better if the patient receives the transplant early versus later in the disease course. 2. Reduced-intensity conditioning (RIC) has improved transplant outcomes for older patients with AML and those with pre-existing co-morbidities. 3. I have the information I need to understand when a patient with AML should be referred for transplant consultation, including the prognostic impact of cytogenetic and molecular markers. 4. I have the information I need to compare the outcomes of a new agent/therapy against transplant for AML. |

**Supplemental Table 5A: Shared Questions in Post-transplant Care Across 2015, 2019, and 2024 Surveys**

| **IV. Post-transplant Care** | **Year Comparison** | | |
| --- | --- | --- | --- |
|  | **2024** | **2019** | **2015** |
| 1. Who provide post-transplant care | x | x | x |
| 2. Adequate training | x | x | x |
| 3. Have information to provide post-transplant care | x | x | x |
| 4. Interest in learning more | x | x | x |
| 5. Distance to the nearest transplant center | x |  | x |
|  | | | |
| 1. After the first 3 months of post-transplant, who primarily provides post-allogeneic transplant care for your patients? (Please select all that apply.)  - I provide post-transplant care - Transplant center - Primary care physician - Other, please specify - I am not sure who is involved in post-transplant care [EXCLUSIVE] | | | |
| Please indicate how strongly you agree or disagree with the following statements regarding post-transplant care. Strongly disagree (1), 2…9, Strongly agree (10). 2. I have had adequate training in post-transplant care.  3. I have the information I need to provide post-transplant care for patients  4. I am interested in learning more about post-transplant care. | | | |
| 5. Distance to the nearest transplant center | Approximately, how far is your practice from the nearest transplant center? Please provide your best estimate.  ___________ miles [RANGE 0 – 999] | None | How far from your primary office (in miles) is the transplant center to which you primarily referred patients? - Transplant is performed at my center - 0-25 - 26-50 - 51-100 - 101-250 - More than 250 |

**Supplemental Table 5B: Non-Shared Questions in Post-transplant Care Across 2015, 2019, and 2024 Surveys**

| **Non-shared Questions in 2019** | 1. How often do you receive an individual care plan from the transplant physician and/or a patient’s transplant care team to guide follow-up care?   - Always  - Often  - Sometimes  - Rarely  - Never   1. What are your biggest challenges in treating post-transplant patients? [Open-ended] |
| --- | --- |
| **Non-shared Questions in 2015** | 1. How many transplant centers have you referred patients to within the last 2 years? [Range: 0-5] 2. How would you describe your overall working relationship with the transplant center to which you primarily referred patients?   Very poor 1, 2…9, Excellent 10   1. Please rate the transplant center to which you primarily referred patients within the last 2 years, on each of the following: Very Poor 1, 2...9, Excellent 10   - Provides me with advice on which patients are the best candidates for transplant  - Is prompt in responding to my questions before transplant consultation  - Keeps me informed of a patient’s status following referral  - Is prompt in responding to my questions following a patient’s transplant  - Provides appropriate patient care and therapies  - Works proactively with me to determine appropriate post-transplant care  - Provides a smooth process for patient referrals  - Provides me with feedback on individual patient cases |

**Supplemental Table 6A: Shared Questions in Demographics Across 2015, 2019, and 2024 Surveys**

| **VI. Demographics** | **Year Comparison** | | |
| --- | --- | --- | --- |
|  | **2024** | **2019** | **2015** |
| 1. Time in clinical care | x | x | x |
| 2. Number in practice | x | x | x |
| 3. Area of practice | x | x | x |
|  | | | |
| 1. What percentage of your professional effort is spent in each of the following capacities?  - % Providing clinical patient care - % Administrative tasks - % Research activities | | | |
| 2. How many hematology and/or oncology physicians are in your practice/institution?  - I have a solo practice - 2-5 physicians - 6-10 physicians - More than 10 physicians | | | |
| 3. Which category best describes the area in which your practice/institution is located?  - Rural/township - Small town - Suburban - Urban | | | |

**Supplemental Table 6B: Non-Shared Demographic Questions in the 2024 Survey**

| **Non-shared Questions in 2024** | 1. Does your practice perform induction treatment for acute leukemia? (Yes or No) 2. Is your practice attached to or part of a dedicated bone marrow transplant (BMT) program within the same system? (Yes or No) 3. Among the patients with hematological malignancies whom you personally treat, approximately what percentage belong to the following racial categories? Please provide your best estimate.   - White or Caucasian  - Black or African American  - American Indian or Alaska Native - Asian  - Native Hawaiian or Pacific Islander  - Other (Specify)  - Prefer not to answer   1. For approximately what percentage is their primary language? Please provide your best estimate.   - English  - Spanish  - Another language  - Prefer not to answer   1. Approximately what percentage fall into each of the following age groups? Please provide your best estimate.   - Under 18 _____%  - 18 – 34 _____%  - 35 – 59 _____%  - 60 – 69 _____%  - 70 – 79 _____%  - 80+ _____%   1. Approximately, what percentage of your patients with hematological malignancies have each of the following types of primary health insurance? Please provide your best estimate. Enter percentage.   - Commercial insurance through employer  - Medicare Parts A & B (physicians and hospitals)  - Medicaid  - VA/Tricare  - Individual commercial insurance policy  - Insurance through health insurance exchange (i.e. through the Affordable Care Act)  - Other insurance (Specify)  - No insurance/cash paying  - Prefer not to answer   1. Approximately what percentage of your patients with hematological malignancies would be classified as low-income (living in poverty)? 2. To which gender identity do you most identify?   - Female  - Male  - Transgender or non-binary  - Not listed / Other  - Prefer not to answer   1. Which of the following best describes your race/ethnicity? Select all that apply.   - White or Caucasian  - Black or African American  - American Indian or Alaska Native  - Asian  - Native Hawaiian or Pacific Islander  - Hispanic or Latino  - Other (Specify)  - Prefer not to answer |
| --- | --- |

**Supplemental Table 7A: Thematic Overview of Tools and Resources Questions in the 2015 and 2024 Surveys**

| **V. Tools and Resources** | **Year Comparison** | | |
| --- | --- | --- | --- |
|  | **2024** | **2019** | **2015** |
| 1. Organization Awareness and opinion | x |  |  |
| 2. Social Media Usage | x |  |  |
| 3. Conferences | x |  |  |
| 4. The need for tools and resources | x |  |  |
| 5. Level of interest in participating in support and educational programs |  |  | x |

**Supplemental Table 7B: Detailed Tools and Resources Questions in the 2024 Survey**

| **1. Organization Awareness and Opinion** | What is your level of knowledge about each of the following?  - Never heard of  - I only know the name  - I am familiar with this organization  A: NMDP  B: Be The Match  C: Center for International Blood and Marrow Transplant Research (CIBMTR)  D: DKMS  E: Leukemia & Lymphoma Society  F: American Cancer Society  G: American Society of Clinical Oncology (ASCO)  H: American Society of Hematology (ASH)  I: Society of Hematology Oncology (SOHO)  J: National Comprehensive Cancer Network (NCCN)  Which of the following best describes your opinion of the above organizations? (Exclude organizations marked as “Never heard of”)  - Very unfavorable  - Somewhat unfavorable  - Neither favorable nor unfavorable  - Somewhat favorable  - Very favorable |
| --- | --- |
| **2. Social Media Usage** | Which of the following social media platforms do you use in a professional capacity to learn about new developments?  - LinkedIn  - Facebook  - Instagram  - X (Formerly Twitter)  - Doximity  - Sermo  - YouTube  - TikTok  - Other (Specify)  - None of the above |
| **3. Conference** | How great is your need for the following tools and resources in your practice?  - 1 (Not at all needed)  - 2  - 3 (Some need)  - 4  - 5 (Greatly needed)  A: Transplant consultation timing guidelines  B: Transplant referral guidelines for patients with comorbid conditions  C: Transplant education materials for patients and family members  D: Training in post-transplant care for you/your staff  E: Post-transplant education materials for patients and family members  F: Financial resources for patients  G: Mental/emotional health resources for patients  H: Caregiver support for patients  I: Transplant outcomes data  L: GVHD-specific education materials  M: Post-transplant screening guidelines  N: Post-transplant vaccination guidelines  O: Individualized post-transplant care plans from the patient’s transplant team |
| **4. The Need for Tools and Resources** | In an ideal world, what tools, resources, or support would increase or streamline access to HCT for your patients? [Open-ended] |

**Supplemental Table 7C: Detailed Tools and Resources Questions in the 2015 Survey**

| **Level of Interest in Participating in Support and Educational Programs** |
| --- |
| Please rate your level of interest in participating in the following types of support and educational programs. (Not at all interested 1, 2...9, Very interested 10)  - Educational program at your practice/institution on transplant and patient care  - Educational program at the transplant center on transplant and patient care  - Educational program online on transplant and patient care  - Educational sessions at national medical conference  - Virtual case review led by transplant physician on transplant and patient care (similar to virtual tumor board)  - Education program delivered by health insurance organization (payer)  - Quarterly brief mailings on transplant advances  - Monthly e-newsletter on transplant advances  - Guidelines on which patients should be referred for consultation, by disease  - Guidelines on GVHD screening and prevention with photo atlas  - Care plan for post-transplant tests and evaluations  - Clear process on how to refer patients to a given transplant center  - Other, please specify. |
| Do you consider the following organizations credible sources for providing physician education on transplant and/or hematologic malignancies? (Not at all credible 1, 2...9, Very credible 10)  - American Society for Blood and Marrow Transplant (ASBMT)  - American Society of Clinical Oncology (ASCO)  - American Society of Hematology (ASH)  - Center for International Blood and Marrow Transplant Research (CIBMTR)  - Clinical Care Options (CCO)  - Leukemia and Lymphoma Society (LLS)  - Medscape  - National Comprehensive Cancer Network (NCCN)  - National Marrow Donor Program (NMDP)/ Be The Match  - Health insurance organizations (payers)  - Other, please specify. |
| In the past 12 months, which of the following were ways you learned about transplant? (Please select all that apply.)  - Attended an online education program  - Attended one or more sessions at a national medical conference  - Reviewed material sent to me in the mail  - Received updates via e-mail newsletter  - Participated in an education program at or sponsored by my transplant center  - Attended an education program either at my institution or local/regional site  - Reviewed online journal articles  - Reviewed information on the Internet or mobile app  - I have not needed to learn information on transplant in the past 12 months  - I have not had time to learn about transplant in the past 12 months  - Other, please specify |
| Please indicate which, if any, you were already aware of as being a program or resource provided by NMDP/Be The Match. (Please select all that apply.)  - Registry of unrelated donors and cord blood units  - Medical education for physicians on transplant  - Physician web site, www.BeTheMatchClinical.org  - Patient web site, www.BeTheMatch.org  - HLA and search consultation  - Transplant outcomes data  - Financial support for patients  - Education and support for patients  - Clinical guidelines for timing of referral for transplant consultation  - Clinical guidelines for post-transplant care  - Newsletter about advances in transplant  - Research conducted through CIBMTR (Center for International Blood and Marrow Transplant Research)  - I am not aware of any of these |

**Supplemental Table 8: Perceptions of Allogeneic Transplant for All Diseases in the 2015 Survey**

| **Current Perceptions of Allogeneic Transplant for All Diseases** |
| --- |
| Please indicate how strongly you agree or disagree with the following statements regarding allogeneic transplants and outcomes of transplants for all diseases. (Strongly disagree 1, 2...9, Strongly agree 10)  - There have been major advances in transplant over the last 5 years.  - Outcomes for transplant have improved over the last 5 years.  - More patients are eligible for transplants than 5 years ago.  - Timing of when patients are referred affects transplant outcomes.  - Reduced-intensity conditioning (RIC) has improved transplant outcomes for older patients and those with pre-existing co-morbidities.  - My patients have had good outcomes from transplants, given their disease.  - The treatments I can provide lead to better outcomes than transplant.  - In general, the benefits of transplant outweigh the risks.  - The chances of finding an unrelated donor have improved in recent years because of larger adult donor and cord blood unit registries. |

**Supplemental Table 9: Results of Non-Shared Demographic Questions in the 2024 Survey**

| **Induction Performed at the Practice/Center** | |
| --- | --- |
| Yes | 70% |
| No | 30% |
| **Attached to Transplant Center** | |
| Yes | 44% |
| No | 56% |
| **Physician Race** | |
| White or Caucasian | 60% |
| Black or African American | 21% |
| American Indian or Alaska Native | 2% |
| Asian | 9% |
| Native Hawaiian or Pacific Islander | 2% |
| Other | 6% |
| **Patient Language** | |
| English | 82% |
| Spanish | 14% |
| Another language | 4% |
| **Patient Age** | |
| Under 18 | 3% |
| 18 – 34 | 11% |
| 35 – 59 | 22% |
| 60 – 69 | 32% |
| 70 – 79 | 23% |
| 80+ | 10% |
| 18+ (Net) | 97% |
| 70+ (Net) | 32% |
| **% of Patients with Low Income** | |
| 0-10 | 23% |
| 11-20 | 27% |
| 21-30 | 20% |
| 31-40 | 14% |
| 41-50 | 7% |
| >50 | 16% |
| **Physicians’ Gender** | |
| Female | 23% |
| Male | 67% |
| Transgender or non-binary | 0% |
| Not listed / Other | 0% |
| Prefer not to answer | 10% |
| **Physicians’ Race** | |
| White or Caucasian | 55% |
| Black or African American | 2% |
| American Indian or Alaska Native | 1% |
| Asian | 26% |
| Native Hawaiian or Pacific Islander | 1% |
| Hispanic or Latino | 4% |
| Other | 2% |
| Prefer not to answer | 13% |
